# Evaluating Geometric Measurement Accuracy Based on 3D Reconstruction of Automated Imagery in a Greenhouse

**DOI:** 10.3390/s18072270

**Published:** 2018-07-13

**Authors:** Jing Zhou, Xiuqing Fu, Leon Schumacher, Jianfeng Zhou

**Affiliations:** 1 Division of Food Systems and Bioengineering, University of Missouri, Columbia, MO 65211, USA; jing.zhou@mail.missouri.edu (J.Z.); fuxiu@missouri.edu (X.F.); Schumacherl@missouri.edu (L.S.); 2 College of Engineering, Nanjing Agricultural University, Nanjing 210031, China

**Keywords:** 3D model reconstruction, structure from motion, geometric accuracy, processing efficiency, high-throughput phenotyping

## Abstract

Geometric dimensions of plants are significant parameters for showing plant dynamic responses to environmental variations. An image-based high-throughput phenotyping platform was developed to automatically measure geometric dimensions of plants in a greenhouse. The goal of this paper was to evaluate the accuracy in geometric measurement using the Structure from Motion (SfM) method from images acquired using the automated image-based platform. Images of nine artificial objects of different shapes were taken under 17 combinations of three different overlaps in *x* and *y* directions, respectively, and two different spatial resolutions (*SR*s) with three replicates. Dimensions in *x*, *y* and *z* of these objects were measured from 3D models reconstructed using the SfM method to evaluate the geometric accuracy. A metric power of unit (*POU)* was proposed to combine the effects of image overlap and *SR*. Results showed that measurement error of dimension in *z* is the least affected by overlap and *SR* among the three dimensions and measurement error of dimensions in *x* and *y* increased following a power function with the decrease of *POU* (*R*^2^ = 0.78 and 0.88 for *x* and *y* respectively). *POU*s from 150 to 300 are a preferred range to obtain reasonable accuracy and efficiency for the developed image-based high-throughput phenotyping system. As a study case, the developed system was used to measure the height of 44 plants using an optimal *POU* in greenhouse environment. The results showed a good agreement (*R*^2^ = 92% and *Root Mean Square Error* = 9.4 mm) between the manual and automated method.

## 1. Introduction

Development of new plant varieties with high yield potential and stress resistance includes identification of plants with better genes and phenotypes. It requires high-throughput and accurate measurements of plant dynamic responses to environmental variations, such as plant height [1], canopy area and other morphological plant parameters [2]. Conventionally plant phenotypes are measured manually in greenhouses and field conditions; this work is highly labor-intensive and time-consuming [3]. Plant phenotyping has become an obstacle for the fast development of new crop varieties and underlies the link between genetic traits and environments [4]. In recent years, various non-contact methods have been developed and tested to accelerate the measurement of plant geometric traits, including photogrammetry [5], Light Detection and Ranging (LiDAR) [1], Time-of-Flight (ToF) camera [6] and Red-Green-Blue-depth (RGB-D) camera [7]. For example, an imaging system was developed in [5] to build two-dimensional (2D) mosaicked orthophotos for the measurement of leaf length and rosette area. Results showed that the relationship between the rosette area and total leaf expansion can be fitted with a power law function. However, the system must be calibrated for distortion to ensure true geometric quantities for measurement and is not capable of measuring plant height due to 2D image. A novel imaging and software platform was developed to measure three-dimensional root traits during the seedling development, and significant differences (*p*-value < 0.05) were detected in morphological root traits of gellan gum-grown plants when grown using hydroponic and sand culture [8]. Meanwhile, a high-precision laser scanning system was used in [1] to reconstruct the architecture of the whole barley plant and take the measurements of the plant geometric dimensions. Results indicated that the laser scanner could estimate the plant height in the decimeter scale and the estimated parameters were highly correlated with the manually obtained parameters (*R*^2^ = 0.85–0.97). However, it is expensive for high-throughput phenotyping using a LiDAR sensor because of the high-cost of LiDAR units and energy consumption [9]. An automatic corn plant phenotyping system was proposed in [6] with a ToF 3D camera. However, spatial resolution (SR) of ToF cameras is very low, thus it tends to be noisy and poorly calibrated for high-throughput phenotyping [10]. Wang [9] introduced a low-cost RGB-D camera system to estimate the size of fruits on trees from an inter row distance of approximately 2 m. The correlation between the manual measurement and machine vision-based estimation was accurate with *R*^2^ = 0.96 and *Root Mean Square Error* (*RMSE*) = 4.9 mm for fruit length and *R*^2^ = 0.95 and *RMSE* = 4.3 mm for fruit width. However, the RGB-D camera performed poorly under direct sunlight, especially for measuring distance information at a large distance (3.5 m and above). Similar to ToF cameras, RGB-D cameras have low resolution in depth, for example, 640 × 480 pixels, at most [11].

Relatively low-cost image-based systems can be useful due to the development of stereovision technology [12]. Structure from Motion (SfM) enables three-dimensional (3D) models to be reconstructed using 2D images acquired from different view angles [13]. Using SfM, the view angles are obtained by moving a single camera around an object of interest [14], which brings the potential of using an easy and low-cost image-based system with a single camera to develop the 3D model of the object [15]. Unlike classic photogrammetric methods which have high requirement in image position and resolution, there is no strict requirement for image overlap and resolution when using SfM based on automated image-based systems (e.g., the scale invariant feature transform (SIFT) of Lowe [16]). The potential of image-based systems for 3D reconstruction and geometric measurement using SfM has been assessed for years in diverse fields, such as surface reconstruction in geoscience [17,18,19], mapping or excavation in archaeology [20,21], and in forestry and agriculture [22,23]. Except for using the Unmanned Aerial Vehicle (UAV) image-based system mentioned above, SfM was also proven to be able to reconstruct fine parts of one or more plants in plant phenotyping. Santos [24] showed that SfM can reconstruct branches and other fine structures of plants, and it took 111 min to process 143 images and 39 min for 77 images using the SIFT method. Jay [14] obtained a strong linear correlation of the estimated plant height (*R*^2^ = 0.99) and leaf area (*R*^2^ = 0.94) with actual values. The measurement errors for height (vertical) and area (horizontal) were *RMSE* = 11 mm, *Mean Absolute Error* (*MAE*) = 0.85 cm, and *RMSE* = 85 cm, *MAE* = 59 cm, respectively. Li [25] established an SfM-MVS (structure-from-motion and multiple-view stereo) system in a greenhouse and able to get depth error of 14.86 mm for object within 1 m distance and of 10 mm for object with less than 800 mm distance.

However, there is no baseline information about the effects of image overlap, SR and camera parameters on geometric measurement accuracy and image processing efficiency using the SfM-based photogrammetric method. Therefore, the goal of this study was to evaluate the accuracy of geometric measurement in plants using the SfM method from sequential images acquired with an automated image-based platform. The specific objectives included (1) to evaluate the effects of image overlap and image resolution on the measurement accuracy and image processing efficiency; (2) to find a balance between processing time and required accuracy; (3) to verify the usability of the proposed method for the measurement of plant height.

## 2. Materials and Methods

### 2.1. Image-Based Phenotyping Platform

**Figure 1:**
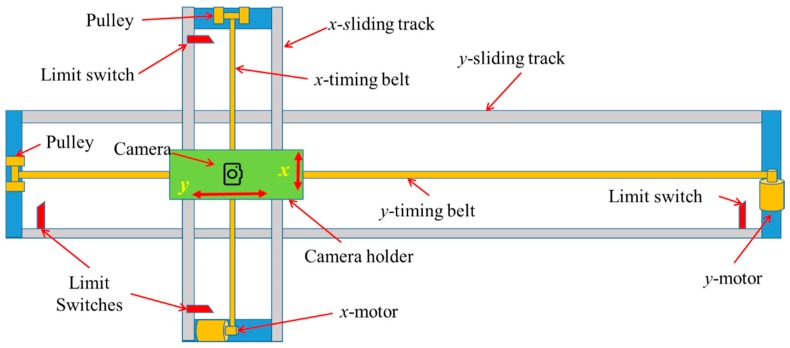
Illustration of the developed image-based platform.

An image-based high-throughput phenotyping platform was developed to automatically take top-view images of plants in a greenhouse. The platform consisted of a frame (7.3 m × 1.7 m) built by two sets of aluminum sliding tracks (STA-BP250, Spokane Hardware Supply, Spokane, WA, USA), two stepper motors (23HS30-2804S, StepperOnline, Nanjing, China), two sets of timing belts (2 mm pitch, 6 mm wide) and motor pulleys (20 teeth with 16 mm diameter), four limit switches (MX-11, Sparkfun Electronics, Boulder, CO, USA), two DC power supplies (ModelS-360-12, Amazon.com, Seattle, WA, USA), and a camera holder. The camera holder is a rectangle frame (400 mm × 150 mm) built by aluminum solid angles, and a camera was fixed to the frame with a bolt and nut. The details of the architecture and major components are illustrated in Figure 1. The camera holder was driven along the *y*-sliding track (horizontal) by a stepper motor through a timing belt which was supported by a timing pulley. The *y*-sliding track was attached to *x*-sliding track (vertical) using four ball-bearing sliders and was driven by another stepper motor that was mounted directly to greenhouse ceiling, approximately 1.5 m above the test bed. Two sets of sliding tracks and driving systems allowed the camera holder to move at desired speeds and patterns in two perpendicular directions.

The control system consisted of a microcontroller (Arduino UNO R3, Sparkfun Electronics, Boulder, CO, USA) and two stepper motor drivers (TB6600, SMAKN, www.DFRobot.com, Shanghai, China). The microcontroller was programmed to output Pulse Width Modulation (PWM) signal with a 75% duty cycle and a selected frequency according to the required motor moving speed. The PWM frequency was regulated based on the actual average moving speed of the camera that was a fraction of the route length over the actual operation time (given by the microcontroller). The stepper motor in *y* would be activated after power on and drive the camera holder moving along *y*-sliding track at a predefined speed. When the camera holder reached the end, the limit switch mounted at the end of the track would be energized to deactivate the *y*-motor and activate *x*-motor to offset the camera holder to an adjacent. Then the *y*-motor was activated, and *x*-motor was deactivated to start a new row. The cycle would be continuing until completing all the scans when the camera holder would return to its original position.

In this study, a digital camera (SX410, Canon USA, Melville, NY, USA) was used to collect sequential images of nine artificial objects (three different shapes) placed on a plant test bed in a greenhouse at the University of Missouri-Columbia, USA. The camera has a resolution of 20.0 Megapixel (5152 × 3864) with the aspect ratio of 4:3 and was configured to take one image at about every 3 s (theoretically) using an open source firmware Canon Hack Development Kit, (http://chdk.wikia.com). Some of the key specifications of the camera are listed in Table 1. The camera was mounted in the camera holder facing to the target objects at the nadir view.

### 2.2. Experiment Design

#### 2.2.1. Experimental Setup

Experiment was conducted in a greenhouse where the average temperature was 25 °C and relative humidity was 24.2% (during experimental time periods). A set of diffusing curtains (Mainstays Shower Liner, local supermarket) were hung around a wooden tank that was built as a test bed for accommodating plants and other objects to avoid direct light spots or shadows on target objects. A digital light sensor (Adafruit TSL2591, Adafruit Industries, New York, NY, USA) was used to measure the light intensity inside the curtain. The test bed was covered with a black fiber sheet to allow the image background to be removed easily [26].

**Figure 2:**
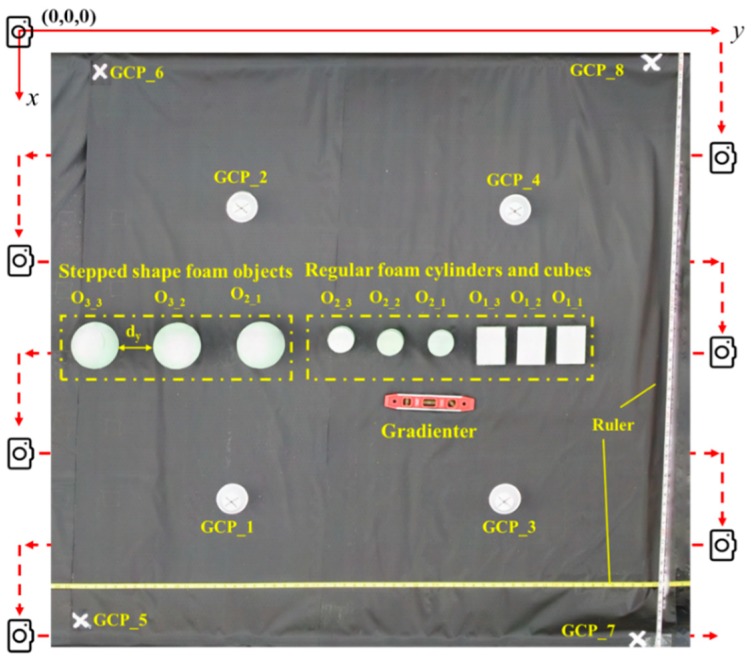
Illustration of experimental setup and imaging pattern.

Figure 2 shows the experimental setup. Different objects were fixed on the black fiber surface and their positions were recorded based on a three-dimensional Cartesian coordinate system where the origin was the starting location of camera route, the positive *x*-axis toward east, the positive *y*-axis toward north and the positive *z*-axis toward up straightly. A gradienter was used to ensure the levelness of the test bed, and two rulers offered reference scale to calibrate the image measurements in *x*- and *y*-dimensions. Ground control points (GCPs) are critical for accurate dimension measurement using the imaging method [27]. In this study, eight GCPs marked by white labels were placed at the four corners of the test bed and in the middle of the imaging area (GCP_1 to GCP_8 in Figure 2) to serve as references for processing a 3D reconstruction model. The coordinates of GCPs were determined by manual measurement using a tape measure.

**Figure 3:**
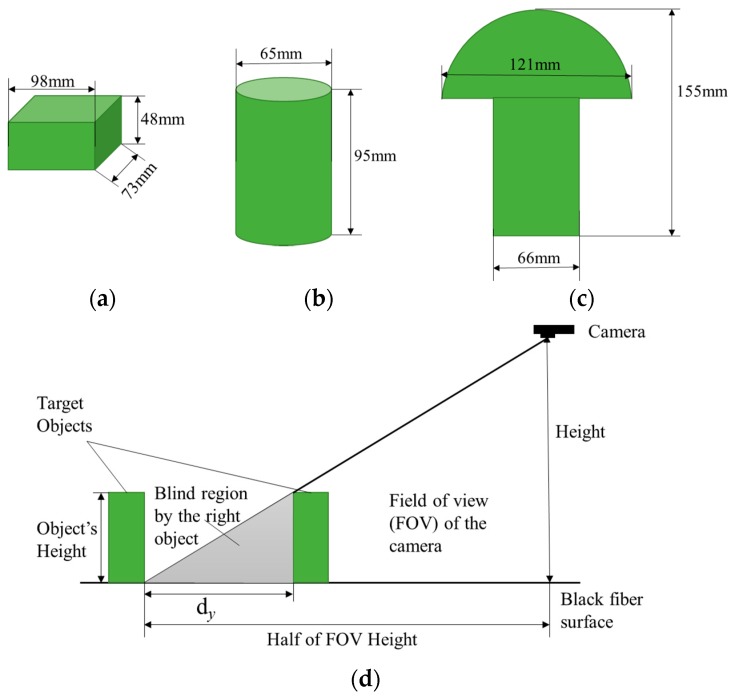
Illustration of the regular artificial objects and their dimensions. (**a**) A cuboid, (**b**) a cylinder and (**c**) a mushroom shape object consisted of a hemisphere and a cylinder. They are abbreviated as O1, O2 and O3 respectively, and three replicates of each object were marked using an underscore with a suborder number, for example O1_1, O1_2, O1_3. (**d**) Calculation of the distance between objects (d*_y_*).

To mimic the different scenarios of plants, three kinds of artificial objects, i.e., cylinders, cuboids, and mushroom shape (Figure 3), were developed using foam materials (FloraCraft^TM^ Dry Foam) and deployed on the top of the test bed. Each object (including replicates) were measured manually three times for each of the three dimensions (*x*, *y*, and *z*) using a caliper. The dimensions shown in Figure 3 are the rounded value of the average measured dimensions. The distance between objects (d*_y_*) was set to avoid blind regions on an object covered by any of its neighbors. Figure 3d shows how the distance was calculated.

#### 2.2.2. Image Overlap and Spatial Resolution

FOV of a camera is defined as the ground area taken into a digital photo, and it is often expressed as dimensions of the ground area, i.e., dimension of long edge (*FOVW*) and of short edge (*FOVH*) of the ground area in an image. *FOV* was calculated using Equations (1) and (2) [28].

(1) $$FOVW=\frac{Sw\times H}{F_{R}}$$

(2) $$FOVH=\frac{S_{h}\times H}{F_{R}}$$

where, *S_w_* and *S_h_* are width and length of camera’s sensor, respectively. *H* is the distance between the camera lens and target area, and *F_R_* is the camera’s focal length. The *FOVW* and *FOVH* of the camera used in this study are listed in Table 1.

The overlap of two images is defined as the percentage of a projected area captured by a camera’s *FOV* from multiple adjacent images (either in *x* or *y* dimension). It is one of the most important parameters for generating accurate 3D models using the SfM method [22,29,30]. Torres-Sánchez [23] also reported the strong underlying control on dense point cloud quality based on the overlap. Image overlap is decided by the *FOV* area of each image and the distance between the centers of the two images in either forward or side directions [28]. Forward (*O_y_*) and side (*O_x_*) image overlap were calculated using Equations (3) and (4), respectively.

(3) $$O_{y}=\frac{FOVH-L_{y}}{FOVH}\times 100\%$$

(4) $$O_{x}=\frac{FOVW-L_{x}}{FOVW}\times 100\%$$

where *L_x_* and *L_y_* were the distances between the centers of two images in side (*x*) and forward (*y*) direction. *L_x_* was determined by the distance between two neighboring routes and *L_y_* was determined by the time interval between two snapshots of the camera and camera’s forwarding speed in *y*. In this study, three different *L_x_* were applied to determine three different side overlaps (Table 2). Different forward image overlaps were obtained by varying the moving speed of the camera. Since the snapshot interval *t_I_* was 3.2 s on average with 0.03 s standard deviation under actual experimental environment, the selected forwarding speeds were set as 22.75, 68.25 and 113.75 mm·s^−1^, resulting in 95%, 85% and 75% forward overlap, respectively. The speed of the snapshot interval was calculated using the collecting time in a route (line) divided by the number of images in this line. Table 2 shows the overlaps and their corresponding number of images. The minimum overlap of images for the reconstruction of 3D point clouds using SfM is recommended as 60% in traditional photogrammetry [31], since the points cloud generated by the overlap with less than 60% cannot provide enough accuracy for dimensional extraction.

Spatial resolution (*SR*) determines the size of the smallest possible feature that can be detected in an image [32]. The relationship between *SR* and image-based problems, such as classification accuracy [33], segmentation scale [34] and quality of photogrammetric measurement [15] have been studied in many fields. *SR* refers to the number of pixel values per unit length (1 mm in this study). *SR* (pixel·mm^−1^) was computed using Equation (5).

(5) $$SR=\frac{imW}{FOVW}$$

where, *imW* is image width (pixel). To evaluate the influence of *SR* on measurement accuracy using the imaging method, dense point clouds of target objects were generated using different *SR*s which were produced by downscaling the original image resolution. For example, a resolution of 5152 × 3864 with a *SR* = 2.78 pixel·mm^−1^ can be downscaled to 2576 × 1932 with a smaller *SR* of 1.39 pixel·mm^−1^. The downscale operation was performed using *imresize* function (Image Processing Toolbox™) in MATLAB (ver. 2016b, The MathWorks, Natick, MA, USA) with default parameters. The nearest-neighbor interpolation method calculates the output pixel value as a weighted average of pixels in the nearest 2-by-2 neighborhood. This is a convenient way to downscale the SR while keeping the other parameters unchanged compared to adjusting the physical height of the camera or changing the camera settings.

As discussed above, higher image overlap results in more common points of two images and provides more references for point registration (a process in point cloud reconstruction to align two point sets). *SR* has a similar effect with image overlap, and both parameters might contribute to the measurement accuracy simultaneously. In real scenarios, image-based dimension measurement might be affected by image *SR*, camera moving speed, mounting height, and route interval. However, the most important factor that determines the measurement accuracy is pixel number in a unit area of an object shared by different images [23]. In this study, a parameter, i.e., power of unit (*POU*), was defined to combine the various factors on the measurement accuracy and was calculated using Equation (6).

(6) $$POU=\frac{SR\times 10^{4}}{\left(100-O_{x}\right)\times \left(100-O_{y}\right)}$$

where, *POU* is the power of unit (pixel·mm^−1^), *SR* (pixel·mm^−1^) is the spatial resolution of individual sequential images, *O_x_* and *O_y_* is the side (in *x*) and forward (in *y*) overlap (%), respectively. In this study, 17 *POU*s of different combinations of *O_x_* and *O_y_* using two different *SR*s are shown in Table 3. The images were collected automatically on three different days. The experiment was carried out from 12 pm. to 3 pm. in each day, when the light condition was from 8000 to 3000 lux.

### 2.3. 3D Dense Cloud Reconstruction and Data Processing

#### 2.3.1. 3D Dense Cloud Reconstruction

The dense point clouds of the target objects were reconstructed based on the SfM method, which is a low-cost photogrammetric method for 3D structure reconstruction from a series of multiple overlapping images. It applies a highly redundant, iterative bundle adjustment procedure, based on a database of features automatically extracted from the set of multiple overlapping images to resolve the target’s structure [35]. The method has been integrated by a range of cloud-processing software, such as Agisoft PhotoScan Pro or Pix4D, which can make direct use of user-uploaded and crowd-sourced photography to generate the necessary coverage of a target scene and can automatically generate 3D dense point clouds from these photo sets [19]. Recently, there has been a growing interest in using these tools to study issues in the field of agriculture, forestry, geoscience, archaeology, and architecture [36].

In this study, sequential images were processed using Agisoft PhotoScan Pro (v1.3.4, St. Petersburg, Russia) running on a desktop PC (Dell Optiplex 5050). The PC was configured as Intel(R) Core i7-7700 CPU (8 cores), 16GB RAM memory, 512 GB SSD hard drive. The protocol of dense cloud processing involves three stages: (1) importing sequential images and geo-reference file; (2) aligning images and adding GCPs (markers); and (3) generating dense points. The parameters were set as “High” with Generic and Reference preselection for image alignment, “High” for reconstruction parameter and “Moderate” for filtering mode. The geo-reference file included location information of each image (camera) that was calculated using accumulated distance intervals from the origin in the established coordinate system (Figure 2). After the imported images were aligned, Agisoft searched for similar features in the images to create a 3D dense point cloud to calculate the dimensions of target objects.

#### 2.3.2. Object Segmentation

The developed dense point clouds were imported to MATLAB and processed based on its Image Processing Toolbox and Computer Vision System Toolbox. The dense clouds were visualized, and individual objects were segmented manually using the function *getrect* in MATLAB before further processing. The function captures vertex coordinates of the rectangle selected manually by users and all the points within the area of these four vertexes were segmented out.

#### 2.3.3. Object Height Correction and Calculation

**Figure 4:**
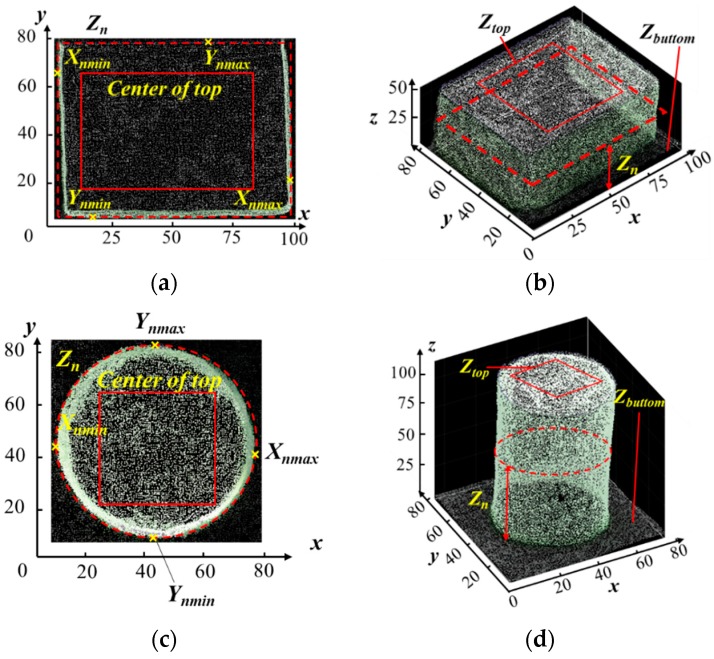

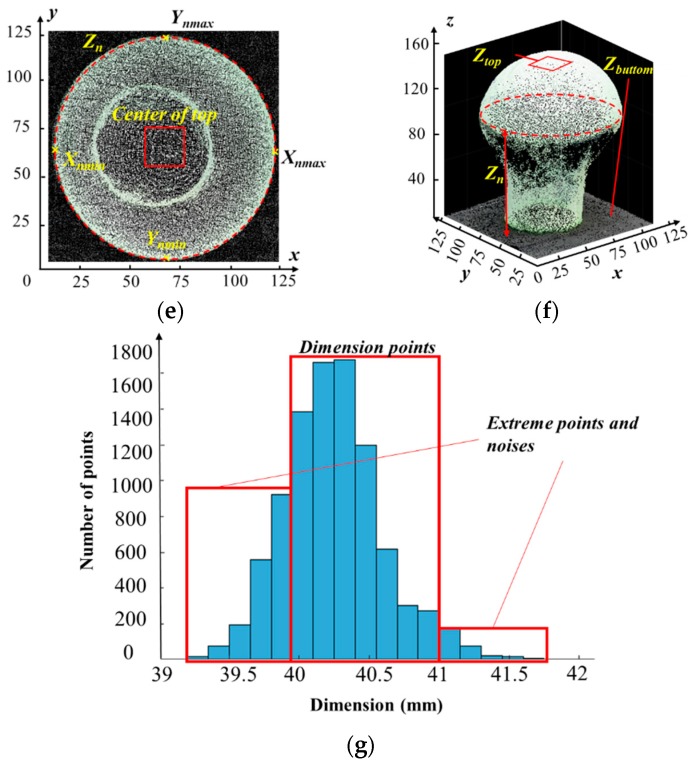
Illustration of the dimension measurement approaches using dense point clouds. Subfigures (**b**,**d**,**f**) are the 3D images of O1_1, O2_1 and O3_1, respectively. Subfigures (**a**,**c**,**e**) are the projected images in *x*-*y* plane and (**g**) shows the way to select dimension points and remove extreme points and noises. The *x*, *y* and *z* dimensional units used in (**a**–**f**) are mm.

The starting location of the camera was set as the origin of the platform leading to negative z coordinates of all the objects below the camera based on the set coordination system. To simplify the height calculation, the z coordinates of the bottom (*Z_bottom_s*) of each individual object were set to zero and, therefore, the average z coordinates of the top (*Z_top_s*) of each object represented the height of the object. Figure 4 illustrates the dimension measurement approaches for three types of objects used in this study. To extract the *Z_top_s* of each object, the center part was manually selected as shown in Figure 4a,c,e. *Zs* of the selected points were divided into 20 bins using histogram counting (*histcounts*) function in MATLAB, and then five of the largest and five of the smallest bins were removed to get rid of the extreme points and noises as shown in Figure 4g. Meanwhile, the dimensions in *x* and *y* were also calculated using point cloud of segmented objects with the following procedure: First, the extreme points in *x* and *y* were selected from a group of points with a same Z. As illustrated in Figure 4a,c,e, four extreme points X_nmin_*,* X_nmax_*,* Y_nmin_ and Y_nmax_ were selected from the group of points with the same Z (marked as Z_n_), thus dimension in *x* at Z_n_ was the difference between X_nmin_ and X_nmax_, and in *y* was between Y_nmin_ and Y_nmax_. Extreme points and noises of the dimensions in *x* and *y* at all *Zs* were removed based on the histogram in Figure 4g.

### 2.4. Accuracy Assessment and Data Analysis

Measurement errors were defined as the absolute of differences between measured dimensions using the imagery method and manual measurements (ground truth). Meanwhile, data processing time for each data set recorded by Agisoft PhotoScan was extracted to compare their time consumed for each dataset. The processing time for depth maps and 3D model reconstructing was reported separately. All statistical analysis was conducted using software SAS 9.4 (SAS Institute, Cary, NC, USA). An analysis of variance analysis (ANOVA) was conducted to compare the difference in the least square means of measurement error due to different setups using “PROC GLM” with the “LSMEANS/PDIFF” option at 0.05 level of significance. The regression between measurement errors and *POU*s was performed using “PROC REG” procedure.

### 2.5. Case Study

The performance in measuring plant height using the imaging method was studied using a group of 44 soybean plants. Soybean seeds were sown in a pot and filled with general purpose peat-based growing medium (PRO MIX, Premier Tech Horticulture, Quakertown, PA, USA). The soybeans were transferred to the wooden tank containing salt water solution (120 mM of NaCl) to induce salt stress starting from soybean V1 stage. The plants were scanned with the automated imagery system with *O_x_* = 80%, *O_y_* = 95% and *SR* = 2.78 pixel·mm^−1^ resulting in a *POU* = 278 pixel·mm^−1^. The height of each plant was also measured manually after imaging with a tape measure (1.0 mm resolution). The measurement accuracy was evaluated by comparing image measurements with manual measurements.

Manual selection of the center part of artificial objects was to minimize any potential error brought by automated processing steps, so that the measurement accuracy directly related to the SfM method could be evaluated. However, the manual selection of *Z_top_s* from each object is time-consuming and practically unrealistic when hundreds or thousands of plants are processed. Therefore, an automated method was designed to compute the geometric dimensions of plants automatically. The method applied a K-means clustering classification on the data points of each plant to segment the plant and remove background, including noises around the plant, based on color (greenness) information. The *kmeans* function in MATLAB was used setting the *class* option as two (plant vs. background and noises) with a classifier of *Triangular Greenness Index* (*TGI*), which was calculated using Equation (7) [37].

(7) TGI=−0.5×[0.19(R−G)−0.12(R−B)]

where, *R*, *G* and *B* are the pixel values of *Red*, *Green* and *Blue* channels in the images, respectively. The *TGI* is a vegetation index that is closely related to the chlorophyll content of plants and might be potentially used to separate plants and none-plant materials [37]. In this study, TGIs with positive values represented plants, while those with zeros or negative values were classified as background and noises. Then the *Zs* of the segmented plant were divided into 50 bins using *histcounts* function and the bin with the largest *Zs* were averaged to get the height of the plant.

## 3. Results

### 3.1. Measurement Accuracy in Three Dimensions

**Figure 5:**
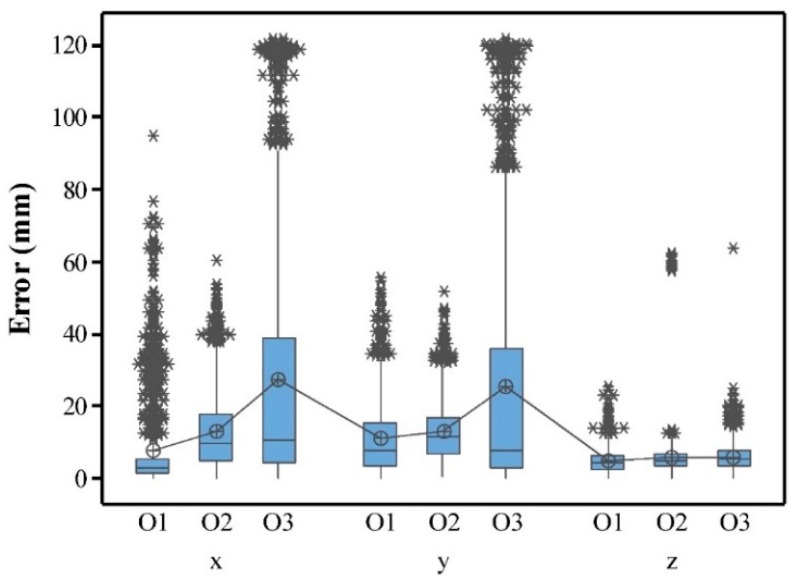
Boxplot of measurement errors in three directions (*x*, *y*, and *z*). In each dimension, the box of each object shape shows errors obtained from three replicates (for example, O1_1, O1_2, O1_3) of a shape under all 17 *POU*s. The circle with a plus sign in each box is the mean of this group. Asterisks above the boxes are outliers in errors.

**Figure 6:**
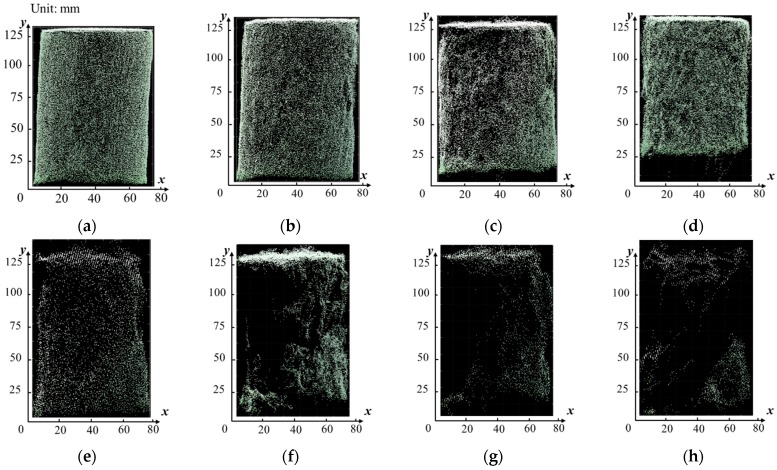
Comparison of the side view of O2 under different *POU*s. Subfigures (**a**–**h**) are the side view of O2 under *POU* of 556, 278, 168, 111, 84, 56, 28, and 17, respectively.

The whole data set of images measured dimensions of nine objects under 17 *POU*s in three replicates are shown in the Supplementary Materials due to the size of dataset. The descriptive statistical summary of the measurement errors in three dimensions and different shapes of objects under 17 *POU*s is shown in Figure 5. The measurement errors varied in a wide range from 0.0 mm to 94.9 mm for O1, 0.0 mm to 62.1 mm for O2 and 0.0 mm to 121.6 mm for O3. The results of ANOVA test (Table 4) indicates that the mean measurement errors in *x* and in *y* of each individual object were significantly higher than that in the *z* (*p*-value < 0.01 at 5% significance level). The possible reason for the lower error in vertical direction might be the more projected points on the top of objects. Figure 6 shows the side view of O2 under different *POU*s. Errors in *x* and *y* were partially due to the incomplete or distorted surface on the side of an object, i.e., right side in Figure 6e and left side in Figure 6f. As a comparison, the top side had more dense points and had less chance of incomplete or distorted surface due to better illustration. Errors in vertical direction were possibly caused by noises, i.e., top side in Figure 6c and bottom side in Figure 6d. Extreme points and noises were removed partially when processing and calculating measurements, leading to a control upon errors, but errors from the lost or wrong displacements of the majority points on surface cannot be ignored.

In scenarios of estimating dimensions of plants, measurement accuracy might be more degraded than measuring artificial objects due to the laminated and occluded structure of plants. Leaves at lower layer, i.e., cotyledon of soybean may lose more points at the tip and edge part than leaves at top layer of the plant, i.e., trifoliate leaves, leading to underestimation of early vigor of plants [38]. Thus, when conducting experiments focusing more on dimensions in horizontal direction, stricter experimental parameters and setups should be considered to achieve higher measurement accuracy. Otherwise, more sophisticated imaging-platforms, such as those providing plant images at side view or zenith view, should be developed to get better performance.

### 3.2. Effect of Object Shape on Measurement Accuracy

The effect of object shape was significant on the measurement errors in *x* and *y* direction (ANOVA, Table 4), where the errors were significantly higher (*p*-value < 0.001) in O3, followed by O2 and O1 (Figure 5). This was due to the hemisphere shape of O3 resulting in less points in the horizontal direction of (*x* and *y*) than those of O1 and O2 (Figure 4b,d,f). A similar finding was seen by [23] that more solid and homogenous surfaces lead to more accurate results.

Measurement errors in *z* were constantly lower than in *x* and *y* in all objects. However, the measurement errors in O2 and O3 were significantly higher (*p*-value < 0.001) than that in O1 (Figure 5). O1, which was in the outer side and had less chance to be shaded, had lower errors in all three directions. Moreover, O1 has the largest top area among all the three objects, leading to more points that were qualified to represent dimension in *z* direction. This “unfairness” is also confirmed by Torres-Sánchez [23] that the reduction of quality in the dense point cloud accuracy did not affect all the objects equally. Objects with more irregular shapes will not have points as sufficient as those with a flattening surface. It is consistent with the phenomenon that some studies with subjects of solid and homogenous surfaces [29] used a lower overlap to achieve accuracy very close to those covered trees with irregular shapes using higher overlap [30].

### 3.3. Relationship between Measurement Accuracy and POU

**Figure 7:**
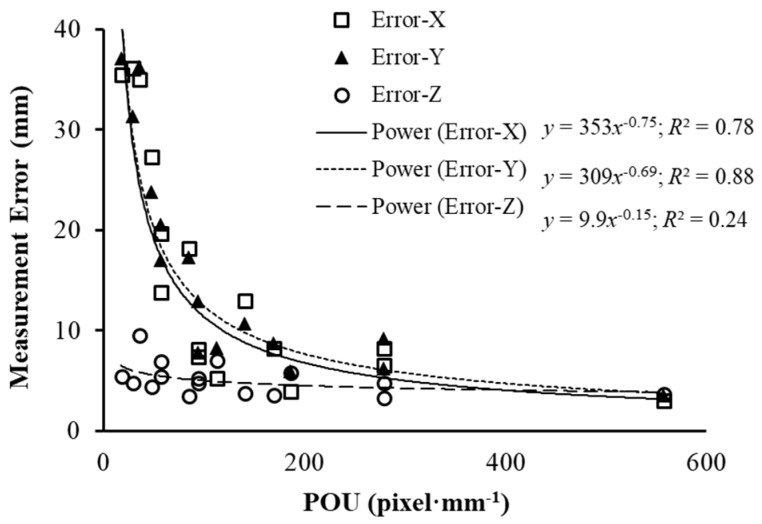
Regression functions between average measurement errors of three shapes and *POU* in three dimensions.

The interaction effect of *O_x_* and *O_y_* was significant on the measurement error, with lower overlap data generating significantly higher error. To quantify the measurement errors due to the *SR* and overlap, a power regression analysis between average measurement errors of the three shapes and defined *POU* was conducted and results are shown in Figure 7. The average measurement errors dropped dramatically with the increase of *POU*. This finding is consistent with the observation by Dandois [22] that the quality of dense point cloud is affected by image overlap and ground sampling distance (GSD), a reciprocal of *SR*. As defined in Equation (5), *POU* is a combination effect of both forward and side overlap and *SR*. High *POU* represented more overlap and higher *SR* of the images. The developed regression functions for measurement errors in *x* and *y* provided a potential tool to determine the imaging quality in greenhouse and field applications where data are collected using the approach similar to this study. It is also identified that measurement errors in *z* were constantly low in this study, which might be caused by the better visibility in top surface than those in the side surface. The projected regions of top side were not decreased as sharply as the regions of the side view. Due to canopy height being an important crop trait that has been widely used [2], it proved a strong support for the usefulness of the proposed data collection method developed in this study.

### 3.4. Relationship between Processing Time and Measurement Accuracy

**Figure 8:**
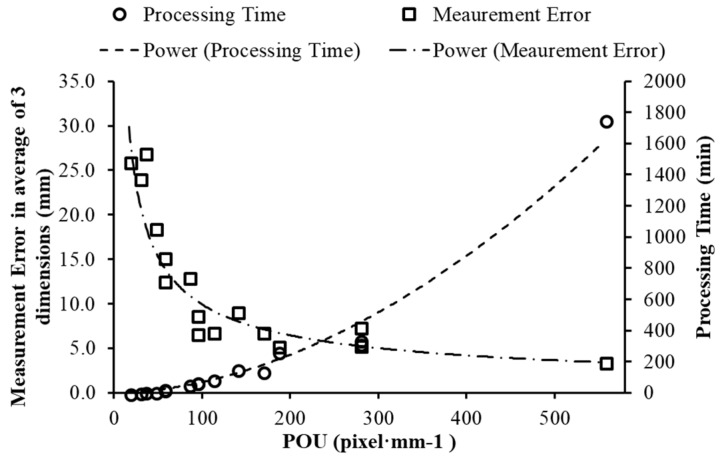
Comparison between processing time and measurement errors. The errors are the averages for all three replicates of all three shapes and all three dimensions, and processing time is based on the first replicate of reconstructing the 3D models.

Average measurement error of the three replicates and the processing time of different *POU*s are shown in Figure 8, which indicates that the measurement error increased, and the processing time decreased with the gradual decrease of *POU*. The processing time in the highest *POU* (556 pixel·mm^−1^ with 90% *O_x_*, 95% *O_y_* and 2.78 pixel·mm^−1^ *SR*) was approximately 1800 min (30 h), which is unrealistic for a high-throughput phenotyping imaging system. The second highest *POU* with 17.6% dense points less than those highest *POU* as shown in Table 5, had an average of 80% less processing time but only 2 mm more errors (Figure 8).

Processing time includes image matching and alignment time as well as depth map and dense cloud generation time. From the lowest *POU* (17) to the highest *POU* (556), the number of images increased from 21 to 292, and the corresponding image matching and alignment time increased from 0.5 min to 38 min, depth map and dense cloud generation time increased from 1 min to 1718 min and the point number for each object increased more than 80% (point number for each object are shown in Table 5). Increasing points resulted from higher overlaps which bring more common points with previous and following images, and from higher *SR* that the region of objects was represented by more individual points. Consequently, it took more time and resources to calculate depth and color information and register this information to point cloud. Therefore, determining *POU* is necessary to balance accuracy and efficiency, leading to an acceptable measurement error without taking an extreme long processing time. A similar conclusion was presented by Torres-Sánchez [23] when considering processing time as affected by image overlap. They also found that the increasing rate in processing time became higher when the overlap was greater than 85%, which agreed with the results in this study. For computers with different performance, processing time may vary, but this curve offered a tendency of comparison between processing time and accuracy, and a reference for later experiments that use similar methods. From Figure 8, *POU* from 150 to 300 can be considered in applications that required error ranging from 8 to 10 mm. The finding was validated using the following case study.

### 3.5. Case Study

**Figure 9:**
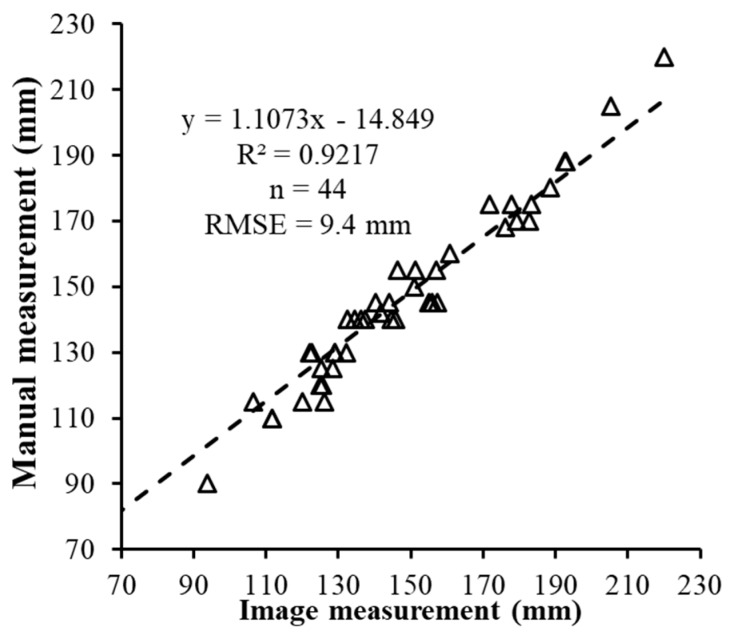
The agreement of image measurements and manual measurements in plant height.

The automated measurement method of height was performed on 44 plants. An ANOVA test (Table 6) shows that there was no significant difference (*p* = 0.907 at the 5% significance level) between the mean plant height measured with a tape measure and the automated measurement from images. Linear regression analysis showed that the automated measurement methods explained 92% of the manual measurements (*R*^2^ = 92%) with a *RMSE* = 9.4 mm. The reason that the error of image measurements for plants was much larger than that of artificial objects might be that artificial objects were easier to be recognized in overlapped images than plant shoots [23], and there were more errors and bias when manually measuring the top point of plant shoots [3], especially when measuring trifoliolates grown in salt-tolerance varieties which were toward one side of the plant obliquely but not right in the center. This can be seen from Figure 9 that measurement in higher plants had larger error than that in shorter plants that only had cotyledon.

Compared with the other studies [14,24,25] introduced previously, this platform can measure geometric dimensions from 3D reconstruction plant model using the SfM method with an acceptable and adjustable error range (5–10 mm). The system may provide not only a high-throughput phenotyping platform for breeding and precision agriculture, but also a user-flexible system that is applicable for different requirements, for example, high demand of accuracy but nonrestrictive processing time, or vice versa.

## 4. Conclusions

Geometric dimensions of plants are significant parameters for showing plant dynamic responses to environmental variations in plant high-throughput phenotyping. Many phenotyping platforms have been developed and applied to measure geometric dimensions using different 3D reconstruction methods in various studies. In this study, a high-throughput phenotyping platform based on nadir point-of-view imaging was developed to automatically measure geometric dimensions of plants in greenhouse using the SfM method. This study mainly focused on evaluating the measurement accuracy of this system. The results proved that measurement errors in three dimensions (*x*, *y*, and *z*) of artificial objects were in the range of 0–121 mm from 3D reconstruction models of 17 different combinations of overlap and *SR*. Measurements in *z* (object’s height) were significantly different with those in *x* and *y*, which had the best accuracy. A metric *POU* was developed in this study to combine the effects of overlap and *SR* on geometric measurement accuracy. The measurement errors in *x* and *y* and *POU* had a power function relationship (*R*^2^ = 0.78 and 0.88 for *x* and *y* respectively). Based on the system setup in this study, *POU* from 150 to 300 is a proper range for obtaining reasonable accuracy and efficiency. A case study of 44 plants were measured using the proposed automated method under *POU* = 278, and it is found that automated measurements had a good agreement with manual measurements (*R*^2^ = 92%, *RMSE* = 9.4 mm).

The goal of this study was to provide an accuracy reference for geometric measurement of plant dimensions using SfM in greenhouse. During data analysis, light effects, object color and the blind region where was covered by neighbor objects to camera in a certain angle were found affecting measurement accuracy. Therefore, future work must include experimental design of different light conditions, object color and distance between neighbor objects.

## Figures and Tables

**Table 1:** Key parameters of experimental setup.

Parameter	Definition/Explanation	Value
*S_w_* (mm)	Camera sensor width	6.17
*S_h_* (mm)	Camera sensor height	4.55
*F_R_* (mm)	Camera focal length	4.00
*imW* (pixels)	Image width	5152
*imH* (pixels)	Image height	3864
*FOVW* (mm)	Width of field of view (*FOV*) at ground level	1851
*FOVH* (mm)	Height of FOV at ground level	1365
*H* (mm)	Distance between camera lens and the test bed	1200

**Table 2:** Overlaps and number of images for processing 3D reconstruction model.

Distance between Two Neighboring Routes *(L_x_*) (mm)	*O_x_* (%)	Forwarding Speed in *y* (mm·s^−1^)	*O_y_* (%)	Number of Images
183	90	22.75	95	330
183	90	22.75	85	119
183	90	22.75	75	81
366	80	68.25	95	156
366	80	68.25	85	61
366	80	68.25	75	43
610	67	113.75	95	106
610	67	113.75	85	42
610	67	113.75	75	30

**Table 3:** *POU* and its corresponding overlap and spatial resolution.

*SR* (pixel·mm^−1^)	*O_x_* (%)	*O_y_* (%)	*POU* (pixel·mm^−1^)	*SR* (pixel·mm^−1^)	*O_x_* (%)	*O_y_* (%)	*POU* (pixel·mm^−1^)
2.78	90	95	556	1.96	90	95	278
2.78	90	85	185	1.96	90	85	93
2.78	90	75	111	1.96	90	75	56
2.78	80	95	278	1.96	80	95	139
2.78	80	85	93	1.96	80	85	46
2.78	80	75	56	1.96	80	75	28
2.78	67	95	168	1.96	67	95	84
2.78	67	85	56	1.96	67	75	17
2.78	67	75	34				

**Table 4:** Results of ANOVA test on the means of errors in three dimensions and each type of object.

Dimension	Means ± Std (mm)		
	O1	O2	O3
*x*	7.8 ± 12.9 ^c^	12.8 ± 10.5 ^b^	27.2 ± 33.7 ^a^
*y*	10.7 ± 10.0 ^d^	13.0 ± 8.2 ^b^	25.4 ± 34.7 ^a^
*z*	4.7 ± 3.2 ^e^	5.5 ± 5.1 ^e^	5.7 ± 3.8 ^e^

Note: The means of error with different lowercase letters had significant difference at 0.05 significance level.

**Table 5:** Number of points for each object using the highest and lowest *POU*.

*POU* (pixel·mm^−1^)	Number of Points of an Object								
	O1_1	O1_2	O1_3	O2_1	O2_2	O2_3	O3_1	O3_2	O3_3
Highest (556)	54,528	52,490	57,283	58,373	60,921	58,715	121,214	120,414	112,502
Lowest (17)	9955	8198	8645	7172	8141	6963	15,314	12,669	14,342

**Table 6:** ANOVA test results of the comparison of image measurements with manual measurements.

	Degree of Freedom	Sum of Squares	Mean Square	*F* Value	*Pr* (>*F*)
**Measurement method**	1	13	12.6	0.014	0.907
**Residuals**	86	78,562	913.5		

## Supplementary Materials

The following are available online at http://www.mdpi.com/1424-8220/18/7/2270/s1.

## References

[1] Paulus S., Schumann H., Kuhlmann H., Léon J. (2014). High-precision laser scanning system for capturing 3D plant architecture and analysing growth of cereal plants. Biosyst. Eng..

[2] Anjum S.A., Xie X.Y., Wang L.C., Saleem M.F., Man C., Lei W. (2011). Morphological, physiological and biochemical responses of plants to drought stress. Afr. J. Agric. Res..

[3] Zhang C., Pumphrey M., Zhou J., Gao H., Zhang Q., Sankaran S. Development of automated high-throughput phenotyping system for controlled environment studies. Proceedings of the 2017 ASABE Annual International Meeting.

[4] Fiorani F., Schurr U. (2013). Future scenarios for plant phenotyping. Annu. Rev. Plant Biol..

[5] An N., Palmer C.M., Baker R.L., Markelz R.C., Ta J., Covington M.F., Maloof J.N., Welch S.M., Weinig C. (2016). Plant high-throughput phenotyping using photogrammetry and imaging techniques to measure leaf length and rosette area. Comput. Electron. Agric..

[6] Chaivivatrakul S., Tang L., Dailey M.N., Nakarmi A.D. (2014). Automatic morphological trait characterization for corn plants via 3D holographic reconstruction. Comput. Electron. Agric..

[7] Remondino F., El-Hakim S. (2006). Image-based 3D modelling: A review. Photogramm. Rec..

[8] Clark R.T., MacCurdy R., Jung J., Shaff J., McCouch S.R., Aneshansley D., Kochian L. (2011). Three-dimensional root phenotyping with a novel imaging and software platform. Plant Physiol..

[9] Wang Z., Walsh K.B., Verma B. (2017). On-tree mango fruit size estimation using RGB-D images. Sensors.

[10] Salinas C., Fernández R., Montes H., Armada M. (2015). A new approach for combining time-of-flight and RGB cameras based on depth-dependent planar projective transformations. Sensors.

[11] Uchiyama H., Sakurai S., Mishima M., Arita D., Okayasu T., Shimada A., Taniguchi R.I. An easy-to-setup 3D phenotyping platform for KOMATSUNA dataset. Proceedings of the IEEE Conference on Computer Vision and Pattern Recognition.

[12] Chéné Y., Rousseau D., Lucidarme P., Bertheloot J., Caffier V., Morel P., Belin É., Chapeau-Blondeau F. (2012). On the use of depth camera for 3D phenotyping of entire plants. Comput. Electron. Agric..

[13] Snavely N., Seitz S.M., Szeliski R. (2006). Photo tourism: Exploring photo collections in 3D. ACM Transactions on Graphics (TOG).

[14] Jay S., Rabatel G., Hadoux X., Moura D., Gorretta N. (2015). In-field crop row phenotyping from 3D modeling performed using Structure from Motion. Comput. Electron. Agric..

[15] Fonstad M.A., Dietrich J.T., Courville B.C., Jensen J.L., Carbonneau P.E. (2013). Topographic structure from motion: A new development in photogrammetric measurement. Earth Surf. Process. Landf..

[16] Lowe D.G. Object recognition from local scale-invariant features. Proceedings of the Seventh IEEE International Conference on Computer Vision.

[17] James M.R., Robson S. (2012). Straightforward reconstruction of 3D surfaces and topography with a camera: Accuracy and geoscience application. J. Geophys. Res. Earth Surf..

[18] Nouwakpo S.K., James M.R., Weltz M.A., Huang C.H., Chagas I., Lima L. (2014). Evaluation of structure from motion for soil microtopography measurement. Photogramm. Rec..

[19] Westoby M., Brasington J., Glasser N.F., Hambrey M.J., Reynolds J.M. (2012). Structure-from-Motion’ photogrammetry: A low-cost, effective tool for geoscience applications. Geomorphology.

[20] Chiabrando F., Nex F., Piatti D., Rinaudo F. (2011). UAV and RPV systems for photogrammetric surveys in archaelogical areas: Two tests in the Piedmont region (Italy). J. Archaeol. Sci..

[21] Sauerbier M., Eisenbeiss H. (2010). UAVs for the documentation of archaeological excavations. Int. Arch. Photogramm. Remote Sens. Spat. Inf. Sci..

[22] Dandois J.P., Olano M., Ellis E.C. (2015). Optimal altitude, overlap, and weather conditions for computer vision UAV estimates of forest structure. Remote Sens..

[23] Torres-Sánchez J., López-Granados F., Borra-Serrano I., Peña J.M. (2017). Assessing UAV-collected image overlap influence on computation time and digital surface model accuracy in olive orchards. Precis. Agric..

[24] Santos T.T., de Oliveira A.A. Image-based 3D digitizing for plant architecture analysis and phenotyping. in Embrapa Informática Agropecuária-Artigo em anais de congresso (ALICE). Proceedings of the Conference on Graphics, Patterns and Images.

[25] Li D., Xu L., Tang X.S., Sun S., Cai X., Zhang P. (2017). 3D imaging of greenhouse plants with an inexpensive binocular stereo vision system. Remote Sens..

[26] Tian L.F., Slaughter D.C. (1998). Environmentally adaptive segmentation algorithm for outdoor image segmentation. Comput. Electron. Agric..

[27] Gindraux S., Boesch R., Farinotti D. (2017). Accuracy Assessment of digital surface models from unmanned aerial vehicles’ imagery on glaciers. Remote Sens..

[28] Canada N.R. (2016). Concepts of Aerial Photography. http://www.nrcan.gc.ca/node/9687.

[29] Turner D., Lucieer A., de Jong S.M. (2015). Time series analysis of landslide dynamics using an unmanned aerial vehicle (UAV). Remote Sens..

[30] Rosnell T., Honkavaara E. (2012). Point cloud generation from aerial image data acquired by a quadrocopter type micro unmanned aerial vehicle and a digital still camera. Sensors.

[31] DeWitt B.A., Wolf P.R. (2000). Elements of Photogrammetry: With Applications in GIS.

[32] Canada N.R. (2015). Spatial Resolution, Pixel Size, and Scale. http://www.nrcan.gc.ca/node/9407.

[33] Chen D., Stow D., Gong P. (2004). Examining the effect of spatial resolution and texture window size on classification accuracy: an urban environment case. Int. J. Remote Sens..

[34] Huang H., Wu B., Fan J. Analysis to the relationship of classification accuracy, segmentation scale, image resolution. Proceedings of the 2003 IEEE International Geoscience and Remote Sensing Symposium (IGARSS’03 2003).

[35] Snavely N., Seitz S.M., Szeliski R. (2008). Modeling the world from internet photo collections. Int. J. Comput. Vis..

[36] Özyeşil O., Voroninski V., Basri R., Singer A. (2017). A survey of structure from motion. Acta Numer..

[37] Hunt E.R., Daughtry C.S.T., Eitel J.U., Long D.S. (2011). Remote sensing leaf chlorophyll content using a visible band index. Agron. J..

[38] Tuberosa R. (2012). Phenotyping for drought tolerance of crops in the genomics era. Front. Physiol..

